# Does depressurization of the portal vein before liver transplantation affect the recurrence of HCC? A nested case-control study

**DOI:** 10.1186/s12885-024-12322-6

**Published:** 2024-05-03

**Authors:** Guo Wei, Yong Zhao, Shifeng Feng, Jingsheng Yuan, Gang Xu, Tao Lv, Jian Yang, Lingxiang Kong, Jiayin Yang

**Affiliations:** 1 Department of General Surgery, Public health clinical center of chengdu, Chengdu, Sichuan Province China; 2 Department of Liver transplantation center, West China Hospital of Sichuan University, Chengdu, Sichuan Province China; 3 Department of Liver transplantation Laboratory, West China Hospital of Sichuan University, Chengdu, Sichuan Province China

**Keywords:** Hepatocellular carcinoma (HCC), Liver transplantation (LT), Tumor recurrence, Portal hypertension (PHT), Portosystemic Shunt (PSS), Splenectomy

## Abstract

**Background:**

Portal hypertension (PHT) has been proven to be closely related to the development of hepatocellular carcinoma (HCC). Whether PHT before liver transplantation (LT) will affect the recurrence of HCC is not clear.

**Methods:**

110 patients with depressurization of the portal vein (DPV) operations (Transjugular Intrahepatic Portosystemic Shunt—TIPS, surgical portosystemic shunt or/and splenectomy) before LT from a HCC LT cohort, matched with 330 preoperative non-DPV patients; this constituted a nested case-control study. Subgroup analysis was based on the order of DPV before or after the occurrence of HCC.

**Results:**

The incidence of acute kidney injury and intra-abdominal bleeding after LT in the DPV group was significantly higher than that in non-DPV group. The 5-year survival rates in the DPV and non-DPV group were 83.4% and 82.7% respectively (*P* = 0.930). In subgroup analysis, patients in the DPV prior to HCC subgroup may have a lower recurrence rate (4.7% vs.16.8%, *P* = 0.045) and a higher tumor free survival rate (88.9% vs.74.4%, *P* = 0.044) after LT under the up-to-date TNMI–II stage, while in TNM III stage, there was no difference for DPV prior to HCC subgroup compared with the DPV after HCC subgroup or the non-DPV group.

**Conclusion:**

Compared with DPV after HCC, DPV treatment before HCC can reduce the recurrence rate of HCC after early transplantation (TNM I-II). DPV before LT can reduce the recurrence of early HCC.

**Supplementary Information:**

The online version contains supplementary material available at 10.1186/s12885-024-12322-6.

## Introduction

At present, liver transplantation (LT) is still the best choice for hepatocellular carcinoma (HCC) patients with liver cirrhosis with a better long-term survival rate and lower recurrence rate of HCC [1, 2]. Gastrointestinal bleeding and refractory ascites caused by portal hypertension (PHT) are the most common complications that occur during the transplantation waiting period [3–5]. Once gastrointestinal hemorrhage occurs, the mortality in Child-Pugh C patients is as high as 40% within the first 6 weeks of bleeding [6]. Depressurization of the portal vein (DPV), not only controls gastrointestinal bleeding, but also reduces ascites relieving abdominal discomfort, and significantly reduces hepatorenal syndrome caused by replenishing insufficient volume [3, 7–9]. DPV practices and preferences vary throughout the world, the main operative methods for DPV include splenectomy and portosystemic shunt and particular radiological DPV. DPV has become a frequently-used bridging therapy for LT [10–14].

Cirrhosis is the most significant independent risk factor for HCC [15, 16]. Although PHT is mainly caused by cirrhosis, it has also been shown to be a risk factor of HCC independent of cirrhosis [17]. In addition, HCC recurrence may be related to PHT in HCC radiofrequency ablation treatment [18]. According to the theory of soil and seeds [19], LT is different from other surgical treatments that remove seeds (HCC). LT completely removes the soil (sick liver) and seeds together, in theory, eliminating the possibility of recurrence. However, 5-year recurrence rates are still 7.8%, and can even reach up to 40% in HCC LT beyond the Milan standard [20]. A 2018 retrospective analysis by Mazzaferro et al. found that the 5-year cumulative mortality rate related to the recurrence of HCC after LT was 8.1%, accounting for 1/3 of the total death of the LT recipients [21]. Therefore, HCC recurrence following LT is still an important research area. Based on the evidence that PHT is closely related to HCC; the purpose of this study was to observe whether reducing the portal pressure limits the recurrence of HCC after LT.

## Methods

This work has been reported in line with the STROCSS criteria [22]. The unique identifying number of this retrospective research is ChiCTR2000032141(date of first registration 20/04/2020, http://www.chictr.org.cn/showproj.aspx?proj=52598). Sichuan University West China Hospital and the Public Health Clinical Center of Chengdu Hospital are cooperative hospitals. The Public Health Clinical Center of Chengdu provides initial outpatient and follow-up services for some liver disease patients, but all surgeries are completed by West China Hospital. The two closely cooperate to form a medical whole, so patients are not distinguished.

### Inclusion and exclusion criteria

The inclusion criteria of the open cohort were as follows: HCC patients 18 years or older without invasion of the main branch of the portal vein or the hepatic vein; HCC that did not directly invade the adjacent organs (except the gallbladder) or penetrate the peritoneum; There was no extrahepatic metastasis (TNM ≤ III A). The patients all had liver cirrhosis and portal hypertension. The diameter of portal vein was measured by ultrasound to determine whether the diameter was increased and to detect liver cirrhosis. Exclusion criteria included retransplantation, multiple organ transplantation, domino LT, double donor LT, or treatment with mTOR inhibitors (e.g., Everolimus/Rapamune) after transplantation. LT grafts were donated by patients who suffered cardiac death (*n* = 494) or brain death (*n* = 298). All the HCC were examined by pathology after undergoing a DPV operation. All included tumors were simple HCC, other tumor types such as cholangiocarcinoma or other special types of liver tumors were excluded. Patients with portal hypertension related complications, such as severe gastrointestinal varices, were informed of the risk of gastrointestinal bleeding and voluntarily decided to undergo DPV treatment.

### Diagnostic criteria and follow up for HCC LT

TNM classification of HCC was conducted following the American Joint Committee of Cancer (AJCC) AJCC Cancer Staging Manual 8th edition in 2017 ( [23]). Acute renal injury (AKI) was assessed using the 2015 edition of the International Club of Ascites: the increase of sCr in 48 h was ≥ 0.3 mg/dl (26.5 μmol/L); or the increase of sCr in 48 h was ≥ 1.5 times of baseline value; or the urine volume lasted for 6 h < 0.5 ml·kg^− 1^·H− 1 [24–26]. Follow-up in the outpatient clinic was conducted routinely. Measurements of alpha fetoprotein and hepatitis B virus deoxyribonucleic acid (DNA), and abdominal ultrasonography were done every 3 months, and a CT scan was performed every 6 months. All hepatitis B virus DNA-positive patients were treated with anti-viral therapy before and after surgery. When intrahepatic recurrence was difficult to ascertain, MRI or contrast-enhanced ultrasonography was performed. Tumor recurrence was determined mainly based on radiographic evidence and/or AFP level. Patients who showed tumor recurrence after surgery were treated with the following options: resection, radio frequency ablation, re-LT, transcatheter arterial chemoembolization, or sorafenib. Patients were monitored until October 2019 or until their death, and their medical records were retrospectively reviewed. Our center requires standard treatment guidelines be followed for HCC patients with elevated HCV RNA. Patients are treated with direct-acting antiviral drugs (DAAS) while waiting for transplantation. If HCV recurrence occurs after transplantation, DAAS should be performed as soon as possible. Additionally, the use of glucocorticoids should be withdrawn as soon as possible after LT, and calcineurin inhibitor maintenance therapy (e.g., tacrolimus) should be minimized. No patients included in this study received any downstage therapy prior to LT. Downstaging refers to methods of treating HCC, such as radiation therapy, chemotherapy, and molecular targeted therapy. Patients with PHT are diagnosed through a comprehensive assessment, which includes a medical history review of liver disease, radiological findings indicative of liver fibrosis or sclerosis, endoscopic identification of gastric varices, and portal vein color Doppler ultrasound revealing portal vein dilatation or collateral circulation formation.

## Results

### Case-control study composition

A total of 792 HCC patients underwent their first LT between September 2007 and January 2022. From these patients, we conducted a propensity score matching (PSM) analysis of 114 patients who underwent DPV (TIPS, surgical portosystemic shunt or/and splenectomy) before LT (32 Transjugular Intrahepatic Portosystemic Shunt-TIPS, 82 surgical portosystemic shunt or/and splenectomy), and 342 matched non-DPV control patients based on their PSM score on the day of LT but prior to surgery (Fig.S1).

### Demographic and disease characteristics

Baseline characteristics of the DPV and non-DPV groups, matched prior to surgery on the day of LT, are shown in Table 1. There was no significant difference between the DPV and non-DPV groups with respect to disease or population characteristics before transplantation.

**Table 1:** Baseline demographic and disease features characteristics on the day of LT

Variables	DPV (*n* = 110)	Non-DPV* (*n* = 330)	*P*
Age^Donor^ (years)	38.5 ± 9.92	39.40 ± 10.58	0.433
Male^Donor^ (%)	87(79.1%)	262(79.4%)	0.946
Age (years)	47.6 ± 7.21	47.61 ± 11.98	0.993
Male (%)	93(84.5%)	279(84.5%)	1.000
BMI (kg/m^2^)	23.04 ± 2.53	22.83 ± 3.05	0.515
sCr (μmoI/L)	76.80 ± 36.40	74.56 ± 24.7	0.469
TB (μmol/L)	47.01 ± 37.64	48.07 ± 66.05	0.873
INR	1.3 ± 0.27	1.3 ± 0.35	1.000
PLT( 10^9^/L)	121.76 ± 67.4	117.80 ± 93.7	0.683
MELD	12.12 ± 3.78	12 ± 5.6	0.834
Child-Pugh	8.19 ± 1.48	7.9 ± 2.07	0.175
HBsAg positive (%)	90(81.8%)	271(82.1%)	0.943
HCV (%)	3(2.7%)	10(3%)	0.871
AFP ≥ 400 ng/mL (%)	62(56.4%)	206(62.4%)	0.259
TNM I(%)	54(49.1%)	172(52.1%)	0.582
TNM II(%)	29(26.4%)	72(21.8%)	0.326
TNM III (%)	27(24.5%)	86(26.1%)	0.753
Preoperative HCC treatment**	50(45.5%)	134(40.6%)	0.372
DCD (%)	69(62.7%)	215(65.2%)	0.645
Waiting LT period	35.93 ± 9.79	34.68 ± 11.99	0.323

*110 DPV and 330 non-DPV were matched in a 1:3 ratio. BMI: Body mass index, sCr: serum creatinine; TB: Total bilirubin; INR: international normalized ratio; MELD: model end-stage liver disease; PLT: platelet; HBsAg: Hepatitis B surface antigen; DCD: donors of cardiac death. ** Preoperative HCC treatment includes hepatectomy, radiofrequency ablation, and transcatheter arterial chemoembolization

### Intraoperative and postoperative outcomes between the DPV and non-DPV groups

**Fig. 1:**
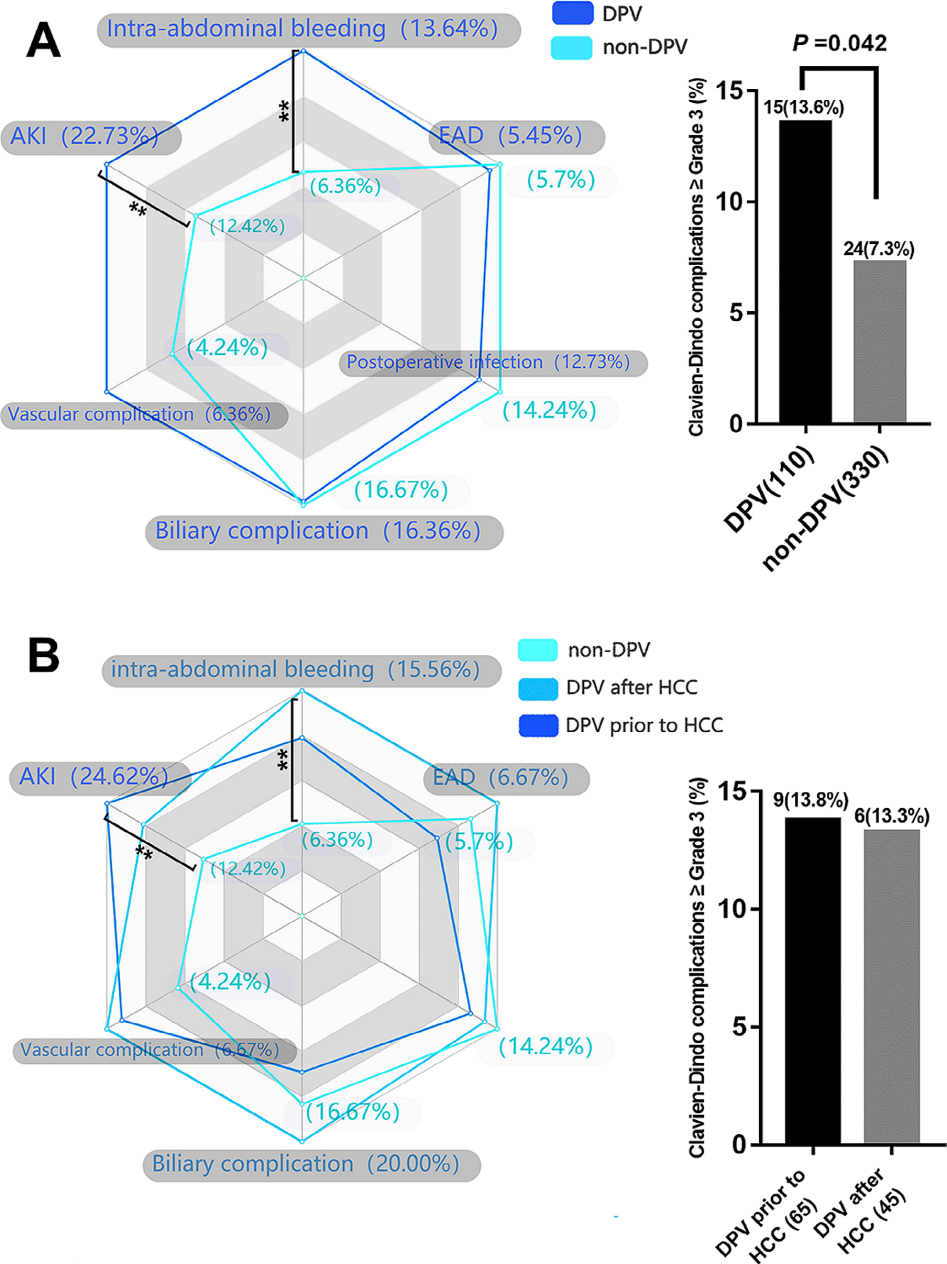
Analysis of main postoperative complications by radar chart and histogram. **P < 0.05 (**A**) The incidence of AKI (22.7% vs. 12.4, *P* = 0.011) and intra-abdominal bleeding (13.6% vs. 6.4%, *P* = 0.012) in DPV group was significantly higher than that in non-DPV group, while the incidence of Clavien–Dindo III-V complication in DPV group was higher than that in non-DPV group. (**B**) In DPV subgroup comparison (DPV prior to HCC and DPV after HCC), there was no significant difference between the two subgroups in the incidence of specific major complications and the incidence of Clavien–Dindo III-V overall complications

**Fig. 2:**
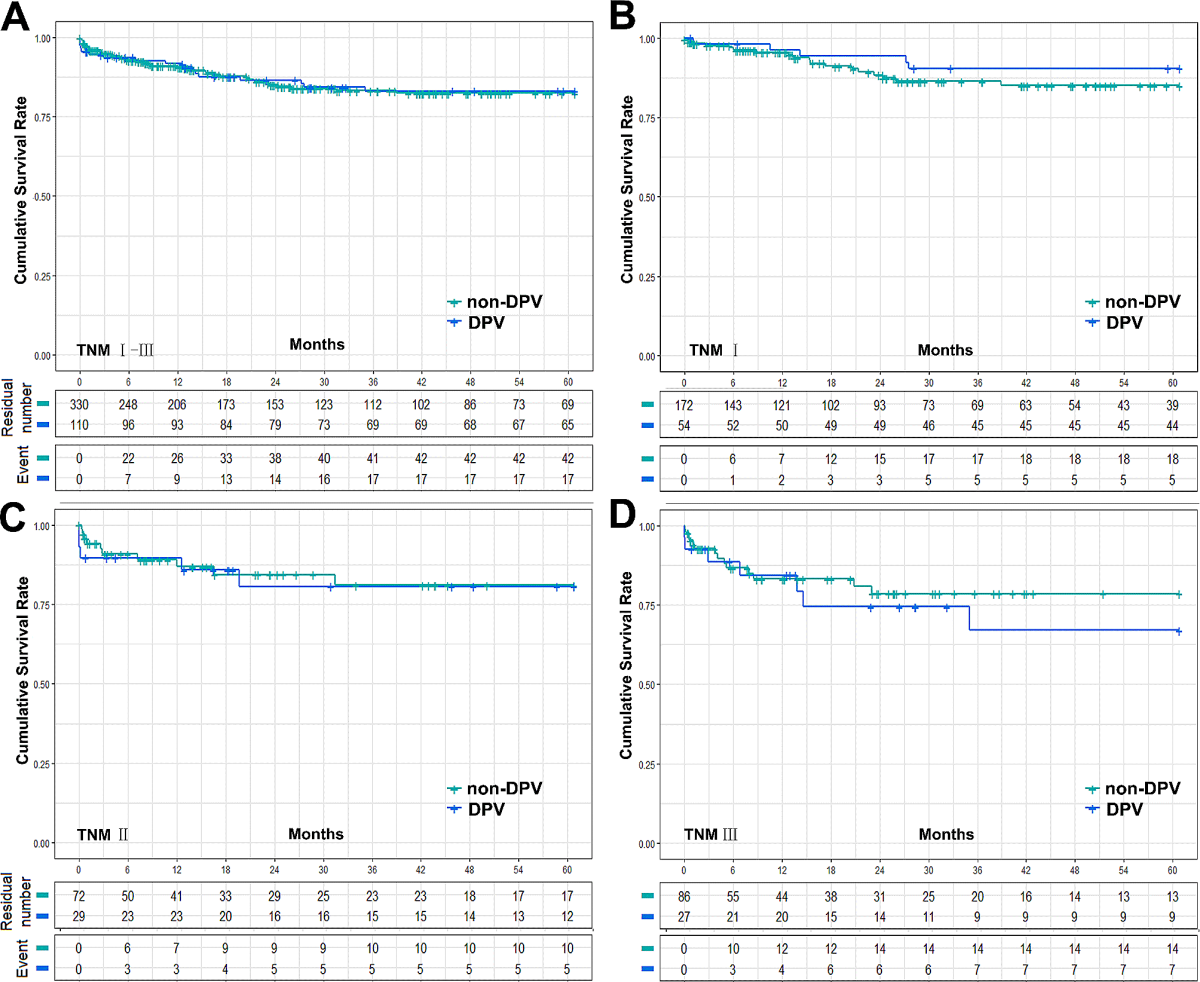
Cumulative survival rate between DPV and non-DPV group. (**A**) The 5-year survival rates of DPV (*n* = 110) and non-DPV (*n* = 330) group were 83.0% and 82.4% respectively, log-rank *P* = 0.934, and the 5-year median survival times were 52.52 ± 1.87 (95% Cl 48.85 to 56.19) and 52.30 ± 1.21 (95% Cl 49.93 to 54.68), respectively. (**B**) In HCC TNMIpatients, the 5-year survival rates of DPV (*n* = 54) and non-DPV (*n* = 172) were 90.4% and 85.1% respectively, log-rank *P* = 0.342, and the 5-year median survival times were 56.55 ± 1.87 (95% Cl 52.89 to 60.22) and 54.18 ± 1.47 (95% Cl 51.31 to 57.05), respectively. (**C**) In HCC TNMIIpatients, The 5-year survival rates of DPV (*n* = 29) and non DPV (*n* = 72) group were 80.7% and 81.0% respectively, log-rank *P* = 0.862, and the 5-year median survival times were 50.59 ± 4.15 (95% Cl 42.45 to 58.73) and 51.28 ± 2.77 (95% Cl 45.85 to 56.72), respectively. (**D**) In HCC TNM III patients, The 5-year survival rates of DPV (*n* = 27) and non DPV (*n* = 86) group were 67.0% and 78.5% respectively, log-rank *P* = 0.483, and the 5-year median survival times were 45.17 ± 5.00 (95% Cl 35.38 to 54.97) and 49.37 ± 2.77 (95% Cl 43.95 to 54.79), respectively

The postoperative complication data for the DPV and non-DPV groups are shown in Fig. 1A. Intra-abdominal bleeding, incidence of postoperative AKI, and Clavien–Dindo Grade III–V complications were significantly higher in the DPV group than those in the non-DPV group. There was no significant difference in cumulative survival rate between the DPV and non-DPV groups within the different TNM stages (Fig. 2).

### HCC and DPV subgroups

**Fig. 3:**
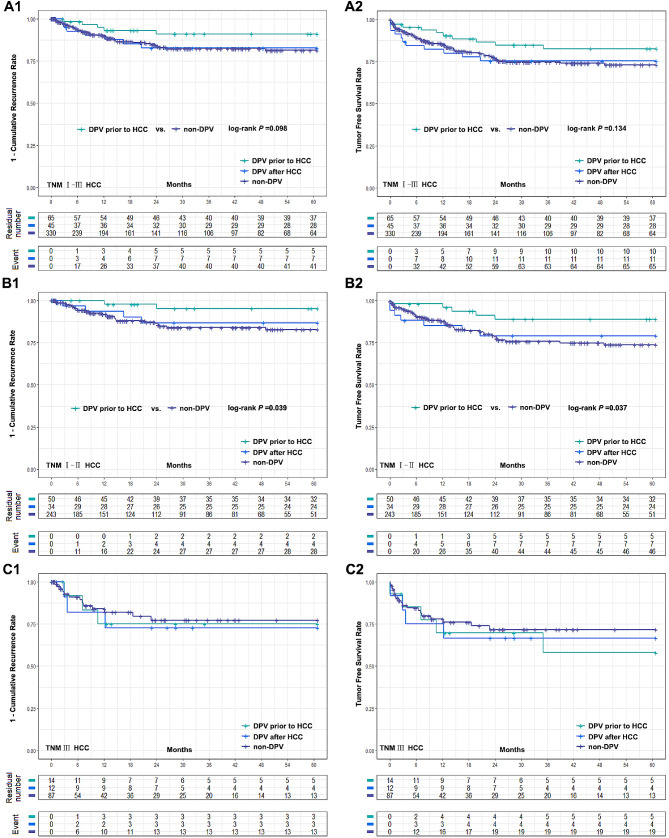
Comparison of cumulative recurrence rate and tumor free survival rate between two DPV subgroups (DPV prior to HCC (*n* = 65) or DPV after HCC (*n* = 45)) and non-DPV (*n* = 330) group after LT. **(A1-A2)** The 5-year cumulative recurrence rate and tumor free survival rate of DPV after HCC subgroup and non-DPV group were 16.4% vs. 18.7%, log-rank *P* = 0.928, 75.3% vs. 73.0% log-rank *P* = 0.979, respectively. The 5-year cumulative recurrence rate and tumor free survival rate of DPV prior to HCC subgroup and non-DPV group were 9.0% vs. 18.7%, log-rank *P* = 0.098, 82.5% vs. 73.0%, log-rank *P* = 0.134. **(B1-B2)** In HCC TNM I-II patients, DPV prior to HCC subgroup were significantly better than non-DPV group in 5-year recurrence rate and tumor free survival rate (4.7% vs. 17.3%, log-rank *P* = 0.039 and 88.9% vs. 73.8%, log-rank *P* = 0.037). There was no significant difference between DPV after HCC subgroup and non-DPV group (13.2% vs. 17.3%, log-rank *P* = 0.619, 79.1% vs. 73.8%, log-rank *P* = 0.671). **(C1-C2)** The 5-year cumulative recurrence rate and tumor free survival rate of DPV prior to HCC, DPV after HCC and non-DPV were 25.0%, 27.3%, 22.8%, and 58.0%, 66.7%, 71.6%, respectively. There was no significant difference between groups (log-rank *P* > 0.05)

DPV patients were divided into two subgroups according to the sequence of their DPV treatment: DPV prior to HCC (*n* = 65) and DPV after HCC (*n* = 45). Subgroup analysis found no significant differences between these groups in postoperative complications (Fig. 1B). However, patients with TNM I–II HCC had a significantly better cumulative recurrence rate and tumor-free survival rate when undergoing DPV prior to HCC compared to the non-DPV group. However, there was no significant difference between the subgroup that received DPV after HCC and the non-DPV group. In TNM III HCC patients, there was no significant difference in tumor recurrence rate or tumor-free survival rate between the non-DPV group and either DPV subgroup (Fig. 3).

## Discussion

At present, HCC recurrence remains the main problem affecting the LT prognosis ( [27]). Although PHT has been shown to be closely associated with the development of HCC ( [28]) and to significantly affect prognosis following other non-LT radical treatments for HCC ( [18, 29, 30]), whether preoperative DPV can affect the prognosis of LT for HCC has not been reported. Our main finding is that patients who underwent DPV prior to HCC had a lower cumulative recurrence rate and higher tumor-free survival rate after LT in the AJCC TNM I–II stages, while in TNM III stage, DPV prior to HCC resulted in no difference between DPV after HCC or non-DPV patients.

The AJCC levels of evidence were established by the AJCC 8th Edition cancer staging system. Most of the confirmed risk factors related to the prognosis of LT for HCC were controlled in the latest TNM stage ( [31, 32]). It is possible that there are relevant HCC risk factors that have not yet been reported due to the uncontrolled nature of even multiple-center studies and the difficulty of performing epidemiological studies on HCC ( [33]). One of the potential limitations of this study is the fact that DPV consists of three different procedures. Although there is no theoretical and clinical evidence suggesting that surgical portosystemic shunt and/or splenectomy will affect HCC recurrence after LT or other radical treatment, TIPS may increase the risk of metastasis caused by intrahepatic shunt through HCC ( [34, 35]). If TIPS after HCC could lead to HCC extrahepatic metastasis, there would be a selection bias in our study. Since LT is not recommended for patients with extrahepatic metastasis, patients who developed extrahepatic metastasis due to TIPS after HCC will be excluded from LT. Wallace et al. reported that 2 of 9 patients developed lung metastases from TIPS crossing through a hepatic malignancy ( [35]). However, Bettinger et al. ( [36]). reported no metastasis was observed in 40 patients with centrally located tumors in the liver (segment VIII, V, and IV). A recent case-control study observing 217 patients found that TIPS is safe for PHT in patients with HCC ( [37]). Although we were unable to confirm that DPV after HCC does not increase the risk of HCC metastasis after transplantation, our results showed there is no increase in HCC recurrence or metastasis in patients who met the criteria for LT and underwent DPV after HCC. Therefore, early LT (before metastasis) for patients undergoing DPV after HCC may prevent this possibility of postoperative potential HCC metastasis.

As early as 1985, Bjørneboe et al. demonstrated that portal-systemic shunt may increase the risk of primary HCC in cirrhosis of the liver based on an observational cohort study of 201 people ( [38]). Banares et al. observed that TIPS may lead to an increase in 5-year cumulative incidence of HCC to 34%, significantly higher than the 25% observed in the control group ( [33]). However, this view has not been unanimously accepted ( [39]). A series of related studies shows that DPV may cause nodular regenerative hyperplasia, but it does not increase the incidence of HCC ( [40–42]) and a recent meta-analysis also found that the incidence of HCC was not increased ( [43]). Moreover, in contrast with other radical treatments, multiple HCC in the liver is not an absolute contraindication for LT. At present, there is still a lack of relevant research reports on this topic. In our study we found no difference between the DPV prior to HCC subgroup and the non-DPV group in the recurrence rate of HCC.

Although we did not observe a difference between patients who received DPV prior to HCC and non-DPV patients in the overall cohort, subgroup analysis highlighted differences between the different tumor TNM stages. Our results found that patients who met the criteria for AJCC TNMI–II stage who underwent DPV prior to HCC had a lower recurrence rate after LT, while patients in TNM III stage, showed no difference between DPV after HCC and non-DPV patients. Relatively low portal pressure may have a protective effect in early HCC patients, but when HCC develops past a certain point this may no longer be effective and this threshold is likely to be between TNM II and III stages. According to the theory by Tanaka et al., portal vein pressure is positively correlated with the pressure of both liver tissue and HCC, and the greater the pressure difference between the tumor and the adjacent tissue, the more likely the tumor is to invade tissue with relatively low pressure ( [44]). Therefore, it is possible that reducing the pressure of the portal vein simultaneously reduces the pressure of the liver tissue. HCC occurs in the liver tissue with relatively normal pressure compared to PHT, which makes the pressure in HCC also relatively low. Therefore, HCC with lower internal pressure is less likely spread into the adjacent tissue. However, with the development of HCC, and an increase in HCC pressure, the pressure difference between the tumor and surrounding tissue begins to be significant. In this scenario, the relatively low pressure of the liver tissue would no longer play a protective role but instead may allow HCC to reach the hepatic vein and enter the circulation easily, eventually leading to an increased risk of recurrence. However, it is worth noting that this interpretation is only a reasonable deduction based on our results and the theory suggested by Tanaka et al. and there is still a lack of direct evidence to support this.

PHT results from elevated intrahepatic vascular resistance due to sinusoidal alterations and liver fibrosis. This condition is compounded by the opening of portosystemic collateral vessels and the formation of new vessels, driven by vascular endothelial growth factor (VEGF) and platelet-derived growth factor (PDGF) ( [45]). HCC, a common complication of cirrhosis, shares similar underlying factors with PHT, increasing the complexity of liver pathology. Inhibition of VEGF receptor 2 signaling has shown potential in reducing collateral vessel formation in experimental models ( [46]). VEGF plays a crucial role in neovascularization and fibrosis within the liver parenchyma, involving hepatocytes and hepatic stellate cells (HSCs). Activated HSCs, stimulated by VEGF, contribute to fibrosis, while portal myofibroblasts aid angiogenesis through collagen production. Liver stiffness influences sinusoidal angiogenesis and fibrosis progression. The resultant intrahepatic neovessels Increased hypoxia, inflammatory cytokines (IL-6, IL-1α), growth factors (epidermal growth factor, transforming growth factor-α and -β, fibroblast growth factor, PDGF) ( [46, 47]). Anti-VEGF therapies like Sorafenib offer promise in mitigating PHT ( [48]). Chronic inflammation and angiogenesis triggered by PHT play a significant role in hepatocarcinogenesis, implying that early intervention targeting angiogenesis in liver disease could hold therapeutic promise.

Since DPV prior to HCC followed by LT can significantly reduce the recurrence rate in the early stage of the tumor, we wondered whether DPV might be applicable to all patients with PHT. However, we are still unsure if early DPV will increase the incidence of HCC and the postoperative complications of DPV are still worthy of consideration. Portal vein thrombosis (PVT) induced by DPV can progress to cavernous transformation, escalating the challenges of LT and amplifying the likelihood of intraoperative and short-term postoperative complications ( [49]). Vigilance is paramount when employing Tips, ensuring they do not penetrate the inferior vena cava excessively, particularly into the right atrium. Misplaced Tips can precipitate severe bleeding and jeopardize patient survival post-LT. Nevertheless, conventional pre-transplant TIPS placement primarily correlates with heightened portal flow gradient and does not significantly impede subsequent LT outcomes ( [50]). . Our study shows that DPV patients had a higher incidence of complications including AKI and postoperative blood loss in HCC stages III–V than non-DPV patients in all TNM stages, but there was no significant difference in the long-term survival rate. At present, there have been few reports about the complications of DPV after LT. Tripathi et al. found that patients who underwent LT after TIPS had higher dialysis rates and the long post-operative hospital stages, but that the overall survival rate is not affected ( [51]). Other associated reports did not focus on postoperative complications, but they all reported that there was still no significant difference in survival between their DPV and non DPV group ( [52]). Therefore, DPV is still a safe and feasible bridging treatment for LT, but it may be accompanied by relatively high complications after LT. It can also be reasonably inferred that the corresponding treatment costs will be increased. Altogether, we believe that it is still necessary to be cautious before deeming DPV applicable to all patients with PHT, especially for those without gastrointestinal hemorrhage or refractory ascites caused by PHT.

The retrospective nature of our work should be acknowledged as a key limitation, even when using a case-control study design based on PSM. However, PSM may lead to other biases due to unmeasured patient characteristics which may have influenced outcomes. Furthermore, because it is difficult to measure, we were unable to directly measure the portal vein pressure. We could only infer a reduction in portal vein pressure through demonstrated methods such as TIPS and obvious postoperative improvement in symptoms. However, we do not know the relationship between the degree of reduction and our results. At present, noninvasive methods of detecting portal vein pressure are indirect and cannot directly reflect the accurate pressure value. Therefore, a technical breakthrough that is able to provide a more in-depth demonstration of the relationship between portal pressure and tumor recurrence is necessary. In addition, our data source was a single center which limits the study’s scope. In HCC TNM III patients, there were only 14 cases of DPV prior to HCC and 12 cases of DPV after HCC. Further multi-center studies are required to verify these findings.

## Conclusion

Based on our results, we propose that non-HCC patients with current DPV indications (such as patients with severe gastrointestinal bleeding and irreversible ascites) should be treated more actively with DPV. If HCC occurred in such patients and LT performed early (TNM I–II), such patients may have relatively lower recurrence rates than patients in non-DPV or DPV after HCC patients. DPV before LT can reduce the recurrence of early HCC.

### Electronic supplementary material

Below is the link to the electronic supplementary material.

Supplementary Material 1

## Data Availability

All related data of our center are stored in the Chinese Liver Transplant Registry, a platform for unified management of LT centers in mainland China (CLTR: http://cltr.cotr.cn). The data that support the findings of this study are available from the Chinese Liver Transplant Registry (CLTR: http://cltr.cotr.cn), but restrictions apply to the availability of these data, which were used under license for the current study, and so are not publicly available. But all related data in this study are available from the corresponding author on reasonable request.
