# Supplementary figures and images for: Does depressurization of the portal vein before liver transplantation affect the recurrence of HCC? A nested case-control study
Source: BMC Cancer. 2024 May 3;24:558. doi: 10.1186/s12885-024-12322-6 (PMC11069182; doi:10.1186/s12885-024-12322-6)

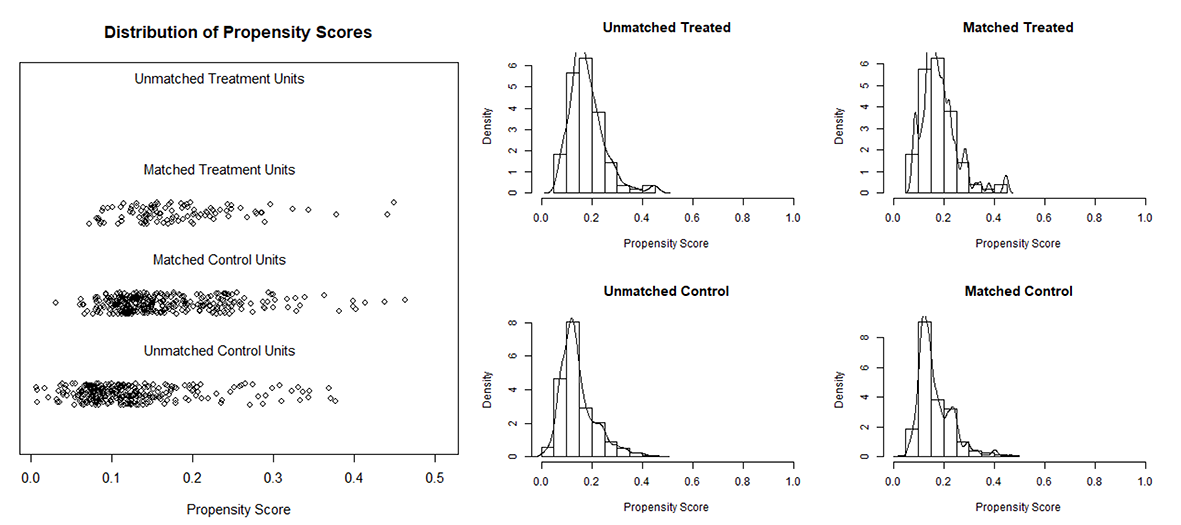
S1. Visualization data for propensity score matching

Supplement: Supplementary file 1 — Supplementary Material 1 [file 12885_2024_12322_MOESM1_ESM.docx]
